# Brief Review of Vibrothermography and Optical Thermography for Defect Quantification in CFRP Material

**DOI:** 10.3390/s25061847

**Published:** 2025-03-16

**Authors:** Zulham Hidayat, Nicolas P. Avdelidis, Henrique Fernandes

**Affiliations:** 1 Integrated Vehicle Health Management (IVHM) Centre, Faculty of Engineering and Applied Sciences, Cranfield University, Bedford MK43 0AL, UK; h.fernandes@cranfield.ac.uk; 2 Department of Aeronautics & Astronautics, School of Engineering, University of Southampton, Boldrewood Innovation Campus, Southampton SO16 7QF, UK; 3 Falculty of Computing, Federal University of Uberlandia, Uberlandia 38408-100, Brazil

**Keywords:** thermography, pulsed thermography, vibrothermography, defect quantification, principal component analysis, variant of principal component analysis

## Abstract

Quantifying defects in carbon-fiber-reinforced polymer (CFRP) composites is crucial for ensuring quality control and structural integrity. Among non-destructive evaluation techniques, thermography has emerged as a promising solution for defect detection and characterization. This literature review synthesizes current advancements in active thermography methods, with a particular focus on vibrothermography and optical thermography, in identifying defects such as delaminations and barely visible impact damage (BVID) in CFRP composites. The review evaluates state-of-the-art techniques, highlighting the advanced applications of optical thermography. It identifies a critical research gap in the integration of vibrothermography with advanced image-processing methods, such as computer vision, which is more commonly applied in optical thermography. Addressing this gap holds significant potential to enhance defect quantification accuracy, improve maintenance practices, and ensure the safety of composite structures.

## 1. Introduction

Composite structures are increasingly utilized across various industries, including aerospace, shipbuilding, and wind-turbine manufacturing, owing to their remarkable mechanical and thermal properties as well as their weight-saving benefits [1,2]. Nevertheless, these composites are prone to damage during both manufacturing and service, with common issues including delamination, fiber breakage, and matrix cracking [3]. Delamination, a prevalent mode of damage propagation characterised by the separation of adjacent layers, can result from improper curing, cutting during manufacturing, or impact from ground service vehicles, tools, and foreign objects during service [4,5]. Research has indicated that visually detecting delamination is particularly challenging, especially in cases of low-velocity impacts that may not present visible damage, highlighting the necessity for advanced inspection methods [5]. Detecting and assessing damage in composite materials is critical to preventing structural failures that could lead to catastrophic events [6]. Damage detection techniques are generally categorized into global and local strategies; while global techniques are effective for identifying the overall presence of damage, local methods excel at pinpointing specific damage locations and assessing its severity [7,8,9]. Non-destructive testing (NDT) is vital in this context, ensuring that damage detection occurs without compromising the integrity of the structures [10]. A thorough identification, quantification, and localization of delamination in composite structures is essential for ensuring safe operational conditions [11]. Several academic journals have explored the application of NDT and assessment methods relevant to composite damage detection [10,12,13,14].

Among the various NDT methods available, infrared thermography (IRT) has gained particular interest due to its ability to provide full-field, non-contact, and rapid defect detection in CFRP structures. Infrared thermography encompasses multiple techniques, including pulsed thermography, lock-in thermography, vibrothermography, laser thermography, eddy-current thermography, and microwave thermography [15]. However, this review focuses exclusively on optical thermography and vibrothermography, as both techniques are widely used for defect quantification in CFRP material but differ in their excitation methods. Pulsed thermography has advanced significantly, integrating this technique with advanced signal-processing techniques such as variants of the principal component analysis (PCA), and deep learning, leading to improvements in defect characterization and quantification [16,17]. In contrast, vibrothermography has seen fewer advancements in image-processing techniques, with most studies still relying on traditional image-processing methods such as traditional PCA, thermographic signal reconstruction (TSR), and pulsed-phase thermography (PPT). However, recent work has introduced manifold learning in low-power vibrothermography, demonstrating its potential for improved defect detection [18]. Despite this progress, such image-processing enhancements remain rare and have not been widely adopted, unlike the more extensive developments seen in pulsed thermography. Existing reviews provide broad overviews of thermographic techniques but lack comparative studies on their image-processing evolution. This review addresses that gap by analyzing the development of both techniques, identifying challenges in vibrothermography, and exploring opportunities for integrating advanced image-processing methods to the low-power vibrothermography.

This literature review examines the state-of-the-art developments in optical thermography and vibrothermography for defect quantification. Additionally, it explores the role of advanced image-processing techniques in enhancing the accuracy of lateral defect detection and quantification. Special attention is given to the application of PCA and its variants, evaluating their significance in improving defect detection and quantification in both vibrothermography and optical thermography. By investigating the implementation of these techniques, this review aims to identify opportunities to bridge methodological gaps and advance the reliability of non-destructive evaluation methods for composite materials.

### 1.1. Factors Affecting the Accuracy of the Defect Quantification

The accuracy of thermographic defect detection in composite materials is influenced by several factors. According to Anwar et al. [19], environmental conditions such as ambient temperature, background reflections, and surface emissivity can significantly impact thermographic measurements. Optimising these parameters is essential for achieving accurate defect detection. Material properties also play a critical role in detection accuracy. A study by Popow et al. [20] found that CFRP often exhibits anisotropic and inhomogeneous thermal properties, which can complicate defect detection. The microstructure of CFRP materials affects heat conduction, leading to challenges in accurately quantifying defects. Additionally, defect characteristics such as size, depth, and thickness influence detection accuracy. Qu et al. [21] found that smaller defects or those located deeper within the material are more challenging to detect due to heat-diffusion losses. Yang et al. [22] further emphasized that deeper defects exhibit lower thermal contrast, making them harder to distinguish from the surrounding material.

Thermal camera specifications play a crucial role in improving the detection and accuracy of defect quantification. Aldave et al. [23] compared three infrared (IR) cameras with varying specifications, which are FLIR SC5000 (MWIR, 320 × 256 pixels, Indium Antimonide), ImageIR 8300 (MWIR, 640 × 512 pixels, Indium Antimonide), and ImageIR 8800 (LWIR, 640 × 512 pixels, Mercury Cadmium Telluride). Specifically, the higher-resolution MWIR camera, the ImageIR 8300 (MWIR, 640 × 512 pixels), achieved the highest defect contrast, particularly for subsurface defects. In contrast, the ImageIR 8800 (LWIR, 640 × 512 pixels) exhibited lower contrast in CFRP due to greater sensitivity to background reflections and limited penetration depth, while the FLIR SC5000 (MWIR, 320 × 256 pixels) struggled with detecting small and deep defects due to lower spatial resolution. The study evaluated these cameras in detecting defects in composite materials (CFRP and Glass Fiber Reinforced Polymer (GFRP)) using active thermography methods, including Optical Pulsed Thermography (OPT) and Optical Lock-in Thermography (OLT). The results showed that camera resolution, spectral range, and detector type significantly impact defect-detection accuracy.

Another significant factor affecting thermography accuracy is noise and outliers in the thermal data. Ebrahimi et al. [24] highlighted that these artifacts can obscure defect signals, leading to undetected defects. To address this issue, advanced image-processing techniques are required to enhance defect detectability. All those factors mention above should be aware when conducting the thermographic testing.

### 1.2. Fabrication of Controlled Defects for Thermographic Inspection

Artificial defects in composite materials are critical for advancing NDT techniques. As defined by Heslehurst et al. [25], a material or structural defect is “any unintentional local variation in the physical state or mechanical properties of a material or structure that may affect the structural behaviour of the component”. Creating controlled defects allows researchers to simulate real-world damage scenarios, enabling the precise calibration and validation of NDT methods. These benchmarks ensure that NDT tools can reliably detect and characterise flaws under actual service conditions [26]. Several research groups have employed different methodologies to simulate defects on composite samples. Liu et al. [27] developed the 12″ × 12″ CFRP coupon as the test specimen by introducing square-shape teflon between specific layers to mimic the delamination defect. A similar methodology was conducted by Andleeb et al. [28], where they developed the carbon-fiber prep-reg laminate sample with a dimension of 450 mm × 500 mm and about 2 mm thick. To achieve artificial delamination, circle-shaped teflon spacers of different diameters were introduced between preferred layers of the sample. This setup allowed for known positions and sizes of the delamination. Shang et al. [29] fabricated test samples from E-glass fiber and epoxy resins. A delamination was fabricated by inserting a piece of teflon tape between the chosen layer. In addition to the delamination, disbound defects are also the object of research interest. To fabricate artificial disbounds, Ptaszek et al. [30] have tapped the hole and inserted a screw with thermally conductive grease. This modification creates a variable-thickness air gap, which more accurately mimics the behavior of real adhesive disbonds under thermographic inspection. Additionally, quartz grains or micro-spacers have been introduced between bonded layers to replicate real-world bonding inconsistencies. Researchers are also using a flat-bottomed hole for defect simulation on the composite material. For example, Wu et al. [31] utilised a GFRP composite board with six machined flat-bottom holes arranged in two rows and three columns. Fernandes et al. [32] used CFRP test specimens that have flat-bottom holes. CFRP is 2.2 mm thick and 148.5 mm × 185 mm in length and width. Barely visible impact damage (BVID) is a type of damage that occurs in CFRP laminates as a result of low-speed impact events, such as dropped tools or debris [33]. It is called “barely visible” because the damage may not be readily apparent to the naked eye, but it can still significantly affect the structural integrity of the laminate [34,35]. To manufacture composite specimens that exhibit BVID, Deng et al. [33] created CFRP specimens with sizes 150 mm × 100 mm × 3 mm. The drop-impact test was performed on each sample using predetermined impact energy levels of 4 J, 6 J, 8 J, 10 J, and 12 J. The impact was executed by the free fall of a hemispherical indenter weighing precisely 2.281 kg. Tan et al. [34] created damage in their CFRP sample using a quasi-static indentation (QSI) by following an ASTM-D6264-17. Katunin et al. [35] introduced low-velocity impact damage with impact energy 3 J, 6 J, and 9 J to their GFRP sample. In conclusion, creating controlled defects in composite materials is essential for refining NDT techniques. These defects allow researchers to simulate real-world damage, ensuring that NDT methods can reliably detect and assess structural flaws.

## 2. Vibrothermography and Optical Thermography Overview

### 2.1. Vibrothermography Overview

#### 2.1.1. Basic Principles of Vibrothermography

Vibrothermography, also known as thermosonics or ultrasound thermography, is an NDT technique initially proposed by Henneke et al. [36]. This method integrates ultrasound excitation with infrared (IR) thermal imaging and is particularly effective for detecting and quantifying damage in composite materials [36].

The fundamental principle behind vibrothermography is that when an object is subjected to ultrasound, it generates vibrations that produce heat at defect sites due to friction and internal damping [36]. This heat is detectable with an infrared camera, allowing for the identification and measurement of the defect’s characteristics [36,37,38].

**Figure 1:**
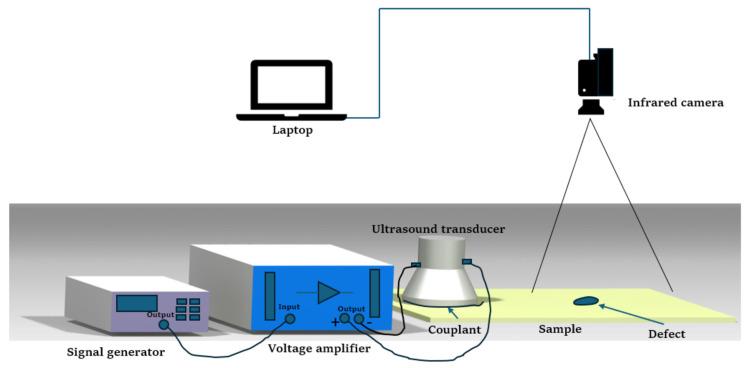
Low−power vibrothermography experiment setup. Image is adapted from Liu et al. [42].

A vibrothermography system typically consists of two main components: an infrared camera for capturing the evolution of the surface-temperature distribution of the specimen and an ultrasound system to generate excitement in the specimen. The system is further complemented by a clamping mechanism and a computer for data analysis [37,39]. To enhance ultrasound transmission, a coupling material is recommended to be placed between the transducer and the specimen, as it not only improves ultrasound transmission but also protects the specimen and prevents misalignment [40]. Experimental findings by [41] revealed that rigidly fixing the specimen at both ends significantly improved crack detection in vibrothermography. This method enhanced the reflection of mechanical waves, thereby converting more mechanical energy into thermal energy at the cracks. Figure 1 provides a typical experimental setup for low-power vibrothermography.

#### 2.1.2. Heat Generation on the Vibrothermography

Rather than employing an external heat source, such as a lamp, as the excitation system, vibrothermography utilises ultrasound or vibration for excitation [36]. The nature of heat generation in vibrothermography has intrigued researchers, prompting them to investigate this phenomenon. According to [43], there are three primary sources of heat generation in vibrothermography: frictional heating, plasticity-induced heat generation, and viscoelastic heating. In vibrothermography, frictional heating occurs through vibration-induced rubbing between crack faces. When high-amplitude vibrations are applied, the crack surfaces rub against each other at small points known as asperities. This intense friction causes heat due to high stress and rapid movements at these points [44]. Additionally, plastic deformations generate heat as cracks propagate, creating thermal energy in the plastic zone beyond the tip of the crack, in conjunction with friction [45]. Viscoelastic phenomena, on the other hand, are particularly relevant to polymeric composites. Many components made of polymeric composites undergo intense vibration during operation [46]. Due to the viscoelastic nature of these materials, stress and strain amplitudes may oscillate out of phase, leading to energy dissipation [46]. Since most polymers have low thermal conductivity, this dissipated mechanical energy translates into heat [46].

Research highlights viscoelasticity as the dominant mechanism contributing to heat generation in disbanding areas of composite plates during vibrothermography tests [47]. Katunin et al. and Wachla et al. [48,49] further proposed a self-heating-based thermography approach, where the specimen is excited at its resonant frequency, generating heat via a viscoelastic effect known as self-heating. Vaddi et al. [50] suggested that vibrothermography can also be used to measure the loss modulus of viscoelastic polymer materials at high frequencies.

#### 2.1.3. Frequency Dependence in Vibrothermography

The excitation frequency plays a critical role in determining the efficacy of this technique [51]. Studies have demonstrated that specific frequencies yield a markedly higher thermal response from defects. For instance, Pieczonka et al. [51] observed that CFRP generates more heat when using a broadband excitation source. A study by Solodov et al. [52] advocated for the utilization of ultrasonic waves excited at the local-defect-resonance (LDR) frequency to enhance defect detection. Applying the LDR frequency facilitates high-contrast imaging within the milliwatt power range [53], potentially eliminating the necessity for high-power ultrasonic excitation [54] and advancing remote ultrasonic thermography via air-coupled ultrasonic excitation [53].

Despite the advantages of using the LDR frequency in vibrothermography, such as reduced specimen damage due to lower power requirements, determining the LDR frequency necessitates additional procedures. Solodov et al. [55] introduced an analytical method to estimate the LDR frequency for flat-bottom holes. The principle involves recognizing that the presence of a defect reduces both local mass and stiffness, resulting in a natural frequency change. When the excitation frequency matches the defect’s local frequency, it induces local defect resonance. The local-defect-resonance frequency ($f_{\mathrm{LDR}}$) derived from the effective stiffness ($K_{\mathrm{eff}}$) and effective mass ($M_{\mathrm{eff}}$) can be expressed as [53,55,56]:

(1) $$f_{\mathrm{LDR}}=\frac{1}{2\pi }\sqrt{\frac{K_{\mathrm{eff}}}{M_{\mathrm{eff}}}}$$

For a circular flat-bottomed hole (FBH) defect with radius *R* and thickness *h*, the effective rigidity and mass are given by [55]:

(2) $$K_{\mathrm{eff}}=\frac{192\pi B}{R^{2}},\;M_{\mathrm{eff}}=1.8\;m\;$$

(3) $$B=\frac{Eh^{3}}{12(1-v^{2})}$$

where *B* is the bending stiffness, *m* is the residual mass of the flat-bottom hole, defined as *m* = *πρh R*^2^, *E* is Young’s modulus, ρ is the mass density of the specimen, and *v* is the Poisson ratio of the material. Using Equations (1)–(3), the resonant LDR frequency for a circular flat-bottom hole can be approximated as:

(4) $$f_{\mathrm{LDR}}\approx 1.6\frac{h}{R^{2}}\sqrt{\frac{E}{12\rho (1-v^{2})}}$$

Alternatively, numerical simulation such as COMSOL version 6.3 Multiphysics [57,58] can be used to determine the local-defect-resonance frequency. However, an understanding of how to simulate the vibrothermography is required. Another method involves employing a laser vibrometer and a broadband piezoelectric actuator to identify all potential resonances within a sample at each point [58,59,60]. Recent numerical simulations by Guo et al. [61] suggest that defect characteristic frequencies may enhance the detection of hidden defects more effectively than the local-defect-resonance frequency, though further experimental validation is required.

In terms of excitation signal application, vibrothermography is primarily conducted using three methods: ultrasound lock-in thermography, ultrasound burst-phase thermography, and ultrasound sweep or modulation frequency thermography [62]. Ultrasound lock-in thermography employs amplitude modulation ultrasound as the excitation [63,64]. Dillenz et al. [65] demonstrated that this technique can detect defects without damaging the part, proving useful in maintenance testing. Its feasibility in detecting hidden corrosion in metal, vertical cracks in ceramic materials, delaminations in wood, and impact damage in CFRP materials has been demonstrated [66].

Ultrasound burst-phase thermography uses short sonic bursts to excite the sample, revealing defects within a narrow frequency range with reduced noise and shorter measuring times [67]. In contrast to single-frequency techniques like ultrasound lock-in and burst-phase thermography, Zweschper et al. [64] proposed using the frequency modulation of a sinusoidal signal, which significantly improves damage detectability by eliminating standing wave patterns. In summary, selecting the appropriate frequency and amplitude signal for the specific material and defect type can enhance detectability and avoid high-power excitation that could harm the specimen.

### 2.2. Optical Thermography Overview

#### 2.2.1. Variants of the Optical Thermography

**Figure 2:**
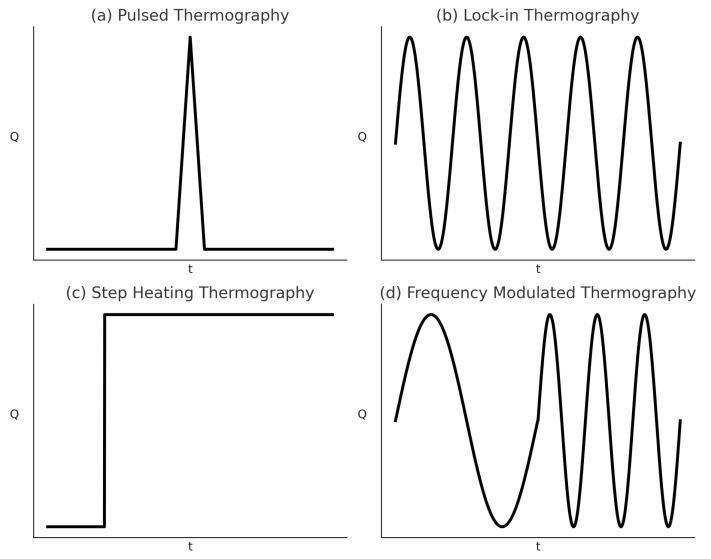
Excitation function of (**a**) pulsed thermography, (**b**) lock-in thermography, (**c**) step-heating thermography, and (**d**) frequency-modulated thermography. Image is adapted from Yang et al. and Ciampa et al. [15,68].

The optical thermography approach varies depending on the kind of external optical heat source employed to generate heat on the sample’s surface and the corresponding heating mechanism. Two common thermal excitation sources are an optical flash or lamp and an optical laser. The optical flash or lamp-based techniques include pulsed thermography (PT), lock-in thermography (LT), step-heating thermography (SHT), long-pulse thermography (LPT), frequency-modulated thermography (FMT), while the optical laser-based techniques consist of laser-spot thermography (LST), and laser-line thermography (LLT) [15,68]. According to Yang et al. [15], optical thermography consists of pulsed thermography, square-pulse thermography, step-heating thermography, lock-in thermography and frequency-modulated thermography thermography. This section will discuss the fundamental principle of common optic-based thermography that is being used to detect damage in the composite which is pulsed thermography, lock-in thermography, step-heating thermography and frequency-modulated thermography. Figure 2 depicts a kind of excitation function signal to conduct pulsed thermography, lock-in thermography, step-heating thermography, and the frequency-modulated thermography experiment.

#### 2.2.2. The Basic Principle of Pulsed Thermography

**Figure 3:**
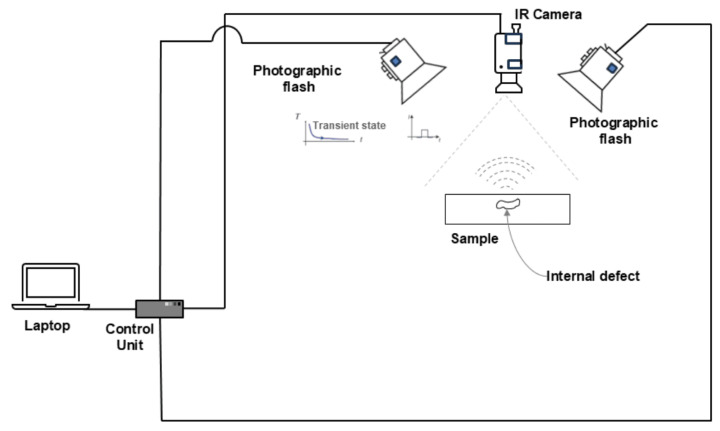
Pulsed thermography experiment setup. Image is adapted from Pereira et al. [72].

Pulsed thermography was first introduced by Parker [69]. Pulsed thermography is a non-destructive evaluation technique that uses a short pulse of heat, often from a xenon flash tube, applied to the material surface, causing heat diffusion that reveals subsurface defects as surface-temperature variations detectable by a thermal imager, such as a scanning infrared camera [70,71]. Figure 3 shows the pulsed thermography experiment to detect a sample with internal defects.

The analysis of the thermal response over time is crucial for identifying and characterising defects [73]. Thermal defect detection relies on heat conduction or diffusion on the solid [73]. By solving the heat-flow equation and considering specific boundary conditions, the variation of the temperature over time on the surface of the specimen can be estimated. The fundamental equation governing this process is the Fourier equation [74,75]:

(5) $$\frac{\partial T}{\partial t}=\alpha \nabla^{2}T$$

where *T* represents temperature, *t* is time, and $\alpha =\frac{k}{\rho c}$. The variable α defines thermal diffusivity (m^2^/s); *k* represents thermal conductivity (W/mK), ρ represents density (kg/m^3^), and *c* represents specific heat (J/kgK). However, the direct application of this equation necessitates several simplifying assumptions. This includes adiabatic conditions (neglecting heat loss), isotropic material properties, and opaque materials, which are often not met in practical situations and lead to inaccuracies [73]. Lau et al. [75] simplify Equation (5) by assuming the specimen is uniformly heated, and the conduction process is regarded as a one-dimensional heat-flow process. The equation becomes:

(6) $$\frac{\partial T}{\partial t}=\alpha \frac{\partial^{2}T}{\partial x^{2}}$$

where *x* represents the depth beneath the material’s surface. From Equation (6), the surface temperature of a semi-infinite half-space specimen after impulse heating or Dirac pulse heating can be determined by this equation [74,76]:

(7) $$T\left(x,t\right)=\frac{Q}{\sqrt{\pi \rho ckt}}e^{-\frac{x^{2}}{4at}}$$

where T(x,t) represents the temperature variation at a depth *x* below the surface at a time *t* following a uniform heat impulse *Q* (J/m^2^) applied at the surface (x=0) at time t=0. The temperature evolution at the surface (x=0) that was scanned by the IR camera will be T(0,t) [76]:

(8) $$T\left(0,t\right)=\frac{Q}{\sqrt{\pi \rho ckt}}$$

#### 2.2.3. Basic Principle of Lock-In Thermography

Lock-in thermography (LT) works by applying a periodic heat source to a specimen’s surface while simultaneously capturing its thermal response using an infrared camera. The periodic heat creates thermal waves that travel through the material, and defects alter the wave propagation by causing phase-delay and amplitude changes in the detected thermal signal. The time delay (phase shift) between the applied heat and the surface response reveals the location and the depth of the defect [77]. Fourier’s law describes the propagation of periodic thermal waves in semi-infinite homogeneous material and can be mathematically expressed as [63,78]:

(9) $$T\left(z,t\right)=T_{0}e^{-\frac{z}{\mu }}\cos\left(\frac{2\pi z}{\lambda }-\omega t\right)$$

where $T_{0}$ is the initial temperature change at the surface of the material, measured in degrees Celsius (°C). The parameter ω=2πf represents the angular modulation frequency, given in radians per second (rad/s), while *f* denotes the excitation frequency of the applied thermal wave, expressed in hertz (Hz).

The thermal wavelength λ describes the spatial cycle of the thermal wave and is given by:

(10) λ=2πμ

The thermal diffusion length μ, which determines how deeply the thermal wave can penetrate before significant attenuation, is expressed in meters (m) and is defined as:

(11) $$\mu =\sqrt{\frac{2\alpha }{\omega }}=\sqrt{\frac{\alpha }{\pi f}}$$

Thermal diffusivity α is a fundamental property of the material that influences the propagation of heat.

**Figure 4:**
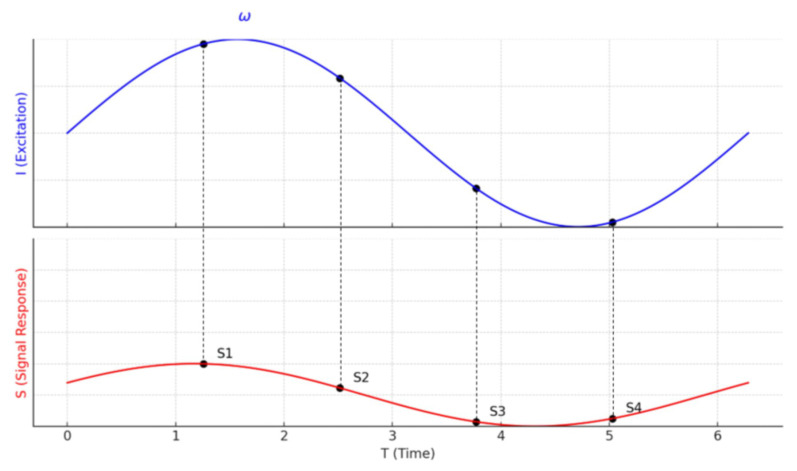
Amplitude and phase extraction from a sinusoidal thermal excitation. Image is adapted from Busse et al. and Castanedo [79,80].

The amplitude and phase delay in lock-in thermography can be determined when the periodic excitation waveform is known. From Figure 4, if both the excitation signal (*I*) and the thermal-response signal (*S*) exhibit sinusoidal behaviour, the amplitude and phase shift can be extracted using four sampled data points per modulation cycle. These values are computed using the following mathematical expressions [79]:

(12) $$A=\sqrt{{\left(S_{1}-S_{3}\right)}^{2}+{\left(S_{2}-S_{4}\right)}^{2}}$$

(13) $$\phi =\arctan\left(\frac{S_{1}-S_{3}}{S_{2}-S_{4}}\right)$$

where *A* represents the amplitude of the thermal response. ϕ is the phase shift of the thermal wave. $S_{1},S_{2},S_{3},$ and $S_{4}$ are four evenly spaced sampling points within one modulation cycle.

#### 2.2.4. Basic Principle of Frequency-/Phase-Modulated Thermography

Frequency-modulated thermography (FMT) involves the use of a frequency-modulated thermal wave to excite the sample. Unlike lock-in thermography, which only uses a single frequency, FMT employs a range of frequencies, allowing for a more comprehensive analysis of the material’s subsurface features [81]. Mulaveesala et al. [82] present an analytical analysis of frequency-modulated thermography to predict the temperature distribution on the surface of the sample, considering the effects of frequency-modulated thermal-wave propagation. Tuli et al. [83] introduced a frequency-modulated thermography method that overcomes the requirement of a high-peak-power heat source, a limitation in conventional pulsed thermography, and lock-in thermography’s limited depth resolution because of a fix regarding the modulating frequency. Rani et al. [84] combine the frequency-modulated thermography and correlation-based pulse-compression approach, significantly enhancing the sensitivity and resolution for detecting deeper defects in CFRP samples compared to conventional data-processing methods. While frequency-modulated thermography offers numerous advantages, it is important to consider the limitations and challenges associated with its implementation. For instance, the complexity of the modulation techniques and the need for precise control over the heating source can pose challenges in practical application.

#### 2.2.5. Basic Principle of Long-Pulse Thermography and Step-Heating Thermography

Long-pulse thermography (LPT) and step-heating thermography (SHT) are the NDT techniques that involve applying continuous heating for a long duration instead of the short, high-intensity pulses used in flash thermography [68]. The key distinction between the two methods lies in their data acquisition approach. In SHT, thermal data are recorded while the heat is being applied, whereas in LPT, the thermal response is captured during the cooling phase, after the heating source has been removed [85]. The application of long-pulse thermography (LPT) is limited due to its susceptibility to noise from environmental radiation and reflections, which can lead to blurry images that hinder accurate defect detection. To tackle the issue of low contrast and blurred edges on the raw data of long-pulse thermography, Wie et al. [86] proposed a novel method for processing infrared image sequences using Fourier transform, phase integration, and edge-preserving filters, which enhances the visualisation of defects in composite materials. Anwar et al. [19] used an uncooled microbolometer and a low-resolution infrared (IR) camera combined with an image-segmentation technique to detect the defect in glass-fiber-reinforced polymer, offering a cost-effective and efficient method for defect assessment with minimal tools. Instead of dealing with artificial damage, Santo et al. [87] attempted to detect real-life damage using the integration of LPT with statistical and machine learning to process the data. A study found that Fuzzy-c clustering outperforms skewness, kurtosis, and PCA in detecting scalpel-blade defects. Step-heating thermography is particularly suited for inspecting thick composite structures, as it allows for deeper defect detection by applying a continuous, low-intensity heat flux over an extended period. A study conducted by Tromaras et al. [88] on the thick monolithic CFRP (20 mm thickness) and sandwich CFRP with the polyethylene terephthalate (PET) foam core. Their findings revealed that defects up to 10 mm depth were detected in a monolithic CFRP sample using SHT, whereas only front skin defects were identified in the CFRP–PET foam–CFRP sandwich component. The challenges of inspecting thick composites using SHT were attributed to low surface emissivity, the significant thickness of the samples, and the thermal diffusion limitations imposed by insulating core materials. A comparison study between pulsed thermography and step-heating thermography conducted by Nategh [89] reveals that step-heating thermography is better suited for material with lower thermal diffusivity such as steel, whereas pulsed thermography application is more effective in a material with higher thermal diffusivity, such as aluminium. Overall, the selection between LPT, SHT, and pulsed thermography should be guided by material properties, defect characteristics, and testing constraints. Future advancements in heating techniques and image-processing algorithms will be crucial for further improving the accuracy and applicability of these NDT methods across a broader range of materials and defect types.

## 3. Damage Quantification by Vibrothermography and Optical Thermography

### 3.1. Damage Quantification by Vibrothermography

This section reviews how vibrothermography quantifies or characterises the damage exhibited in the materials. Damage quantification is a critical step following damage identification and localisation.

Vibrothermography has emerged as a promising method for identifying and characterizing defects, particularly in composite material. Two key studies, those by Zhao et al. [90] and Obeidat et al. [91], propose analytical models to describe the relationship between temperature change and defect depth. While both approaches use 1D heat-diffusion models and validate them through experimental measurements, their methodologies differ in how they extract meaningful parameters from the temperature vs time curve. Zhao et al. [90] directly relate the defect depth to the second derivative of the temperature vs time curve, then applying polynimial fitting to the experimental data to extract depth information. While Obeidat et al. [91] employ a linear fitting approach, correlating defect depth with half-maximum power time (the time at which the temperature reaches half its peak value), peak slope time (the time corresponding to the highest rate of temperature change) and second-derivative peak time (a feature extracted from the second derivative of the thermal curve). Although both studies successfully demonstrate defect depth quantification using a conventional vibrothermography technique, both studies rely on a 1D heat-diffusion model, which simplifies the real-world heat-transfer effect. Both studies also assume that defects have a circular shape, limiting their applicability to more complex defect geometries that are common in real-world composite structures. Below is the analytical model proposed by Zhao et al. [90]:

(14) $$\begin{array}{cc} T(0,t)= & d\sum_{n=-\infty }^{\infty }\left[t^{-\frac{1}{2}}e^{-\frac{g}{t}}-\sqrt{g\pi }+\sqrt{g\pi }\mathrm{erf}\left(\sqrt{\frac{g}{t}}\right)\right]\\ \; & -d\sum_{n=-\infty }^{\infty }\left[t^{-\frac{1}{2}}e^{-\frac{f}{t}}-\sqrt{f\pi }+\sqrt{f\pi }\mathrm{erf}\left(\sqrt{\frac{f}{t}}\right)\right] \end{array}$$

where T(0,t) represents the surface temperature at time *t* above the defect. The material-dependent coefficient *d* is defined as:

(15) $$d=\frac{16\rho c\pi \alpha_{1}}{{\left(4\pi \alpha_{1}\alpha_{2}\alpha_{3}\right)}^{\frac{3}{2}}}$$

where ρ is the density of the material (kg/m^3^), *c* is the specific heat capacity (J/kg·K), and $\alpha_{1},\alpha_{2},\alpha_{3}$ are the thermal diffusivities in the three principal directions (m^2^/s).

The heat-diffusion terms *f* and *g* are expressed as:

(16) $$f=\frac{\alpha_{3}R^{2}+{\left(b-2na\right)}^{2}\alpha_{1}}{4\alpha_{1}\alpha_{3}}$$

(17) $$g=\frac{{\left(b-2na\right)}^{2}}{4\alpha_{3}}$$

where *b* is the defect depth (m), *R* is the defect radius (m), *a* is the sample thickness (m), and *n* represents the thermal-wave reflection index.

A key challenge in vibrothermography is the application of defect-depth quantification using conventional high-power excitation methods. Given that low-power vibrothermography minimizes the thermal impact while maintaining defect detectability, an important question arises: can the existing analytical models be adapted to low-power vibrothermography to quantify defect depth in composite materials? Further investigation is needed to determine whether the same thermal diffusion principles hold under low-energy excitation conditions, potentially expanding the applicability of vibrothermography in the structural-health monitoring of composite materials.

**Figure 5:**
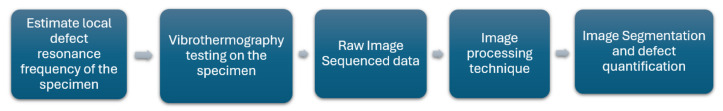
General procedure of low-power vibrothermography defect quantification involving image-processing technique.

Several researchers have attempted to quantify defects by combining low-power vibrothermography with image-processing techniques. Figure 5 illustrates the general procedure for defect quantification, which is commonly used to assess lateral defects in specimens using low-power vibrothermography. This approach has been widely adopted by researchers, including Wei et al. [92]. Their study explores the application of low-power vibrothermography for detecting coating debonds in thermal barrier coatings using a piezoceramic actuator. The study finds that PCA processing yields the highest signal-to-noise ratio (SNR) and temperature contrast, outperforming fast Fourier transform and thermographic signal reconstruction. Their study uses a specimen (Q235 steel substrate with zirconia coating) containing five coating debonds of varying diameters, allowing them to examine how defect size affects detection. However, the study does not consider the variation of the coating thickness and focuses solely on lateral defect detection. It is also required to find out whether low-power vibrothermography can be effectively applied to detect debonds in composite-based coatings, such as CFRP coatings. Huang et al. [93] investigate the application of low-power ultrasonic thermography to detect and quantify artificial delamination in CFRP composites. The authors employ three signal-processing techniques, which are thermal signal reconstruction, PCA, and fast Fourier transform. Similar to the study conducted by Wei et al. [92], the study also found that PCA outperforms the two other signal-processing techniques. The study evaluates how wave-propagation distance, delamination size and defect depth influence detectability. The specimen being used has varying diameter (3 mm to 12 mm) and depths (0.375 mm to 2.25 mm). This controlled approach allows for the detailed investigation of how defect parameters affect detection sensitivity. The study highlights that wave propagation distance influences defect visibility, with defects closer to the ultrasonic source exhibiting higher temperature contrast. Even though the study could capture delamination defects as small as 5 mm in diameter and at a depth of 1.5 mm, investigating the local defect resonance of the specimen beforehand may improve detectability. The study exclusively uses circular-shaped artificial delaminations. Future studies should incorporate variations in the shape of the delamination defect, such as squares, triangles, or irregular geometries. The study conducted by Liu et al. [42] presents a quantitative detection framework for identifying and quantifying corrosion defects in CFRP-strengthened steel structures using low-power vibrothermography. Unlike the study by Wei et al. [92], which used finite-element modeling (FEM) solely to estimate the local defect resonance of the specimen, their study employs FEM both to determine a suitable excitation frequency for the experiment and to simulate heat generation under ultrasonic excitation. The FEM predictions of temperature distribution help optimize experimental conditions, ensuring improved defect detectability. Generally, the defect quantification procedure follows the approach illustrated in Figure 5, with the key contribution being its feature enhancement method, which reduces noise and refines segmentation to improve defect quantification. Additionally, Otsu’s segmentation, similar to the image-segmentation techniques used by Wei et al. [92] and Huang et al. [93], further improves the accuracy of corrosion defect quantification. In their study, the fast Fourier transform and partial least-squares regression algorithm is more suitable compared to the PCA and independent component analysis algorithm in conducting the feature extraction process. It should be noted that this study focuses exclusively on lateral defect detection on the regular shape corrosion (circle shape), without addressing the thickness of the corrosion. It is also needed to develop a simulation of the low-power vibrothermography to the other composite defect, such as BVID, and the delamination defect on the composite material.

BVID is a critical concern in composite structures, as it can lead to significant structural degradation without visible surface damage [34]. One of the main challenges in detecting and quantifying the BVID is that the defect shape and position are unknown beforehand, unlike artificial delaminations or debonds that are pre-defined in controlled experiments. Therefore, validation or a reference method is required to confirm detection accuracy. Several studies have been conducted to quantify this type of defect using low-power vibrothermography. Xu et al. [94] performed a comparative study between low-power vibrothermography and terahertz imaging. The raw image data are processed following the procedure illustrated in Figure 5, where independent component analysis and Fourier transform are applied for image processing, followed by thresholding-based image segmentation. The study reveals that vibrothermography is more effective in detecting deeper defects compared to terahertz imaging. However, the experimental setup does not specify the excitation frequency applied to the three CFRP specimens (with 5 J, 15 J, and 25 J impact damage), making it difficult to replicate the experiment accurately. Zhang et al. [18] introduce an innovative image-processing approach for BVID in CFRP composites using manifold learning. Among variants of manifold learning algorithms, they prefer the local tangent space alignment (LSTA) algorithm in combination with ultrasonic thermography (UT). The study focuses on lateral defect detection without attempting to quantify defect depth. In terms of the validation, while the authors compare their results to pulsed thermography and long-pulse thermography, both methods fail to detect 12 J BVIDs, leaving the LTSA-based ultrasonic thermography approach without a reliable reference for result verification. Another machine learning or deep learning-based image-processing and image-segmentation method for improving defect quantification may need to be investigated.

The study conducted by Liu et al. [95] presents a different approach to detecting BVID by relying on raw thermal image data instead of image-processing techniques for defect detection. The raw image data are obtained using a narrowband sweep-frequency excitation, which enhances defect contrast and improves visibility in BVID inspections. However, the experiment requires laser Doppler vibrometry for pre-measurement, adding complexity to the setup. One of the most interesting aspects of this study is its validation method; rather than using another NDT technique, the authors physically cut the specimen in half to directly inspect the internal structure and confirm the presence of the defect. This direct validation approach ensures defect identification, eliminating uncertainties. A LDR-based vibrothermography study was also conducted by Sharma et al. [96]. Unlike Liu et al. [95], who focus on optimizing sweep duration, their study compares the effectiveness of the forward-sweep excitation and backward-sweep excitation to enhance the flat-bottomed hole and BVID of CFRP detection. The study reveals that backward-sweep excitation leads to higher defect vibration amplitudes and improved thermal contrast. Their study also incorporates laser Doppler vibrometry for LDR frequency measurement, but instead of relying solely on thermal imaging for defect detection, they employ phased-array ultrasound testing (PAUT) as an independent verification method. This additional validation step strengthens the reliability of their findings by confirming defect locations and areas of the defect.

Further advancements in vibrothermography have explored its integration with other NDT techniques. The study conducted by He et al. [97] introduces an innovative approach for detecting BVID in CFRP structures by combining nonlinear ultrasound (NU) and vibrothermography under a shared excitation setup. This integration enhances energy efficiency and detection sensitivity while eliminating the need for separate NDT setups. Additionally, the nonlinear ultrasonic response serves as an independent validation of defect existence by detecting higher-order harmonic components associated with damage. The study employs fast Fourier transform processing to improve the defect visibility. Additional image-processing methods, such as PCA or thermal signal reconstruction, could be explored to further enhance defect contrast. Additionally, image-segmentation techniques could be applied to quantify defect areas and provide more precise defect measurements. Liu et al. [98] introduce an integrated Leaky Lamb Wave (LLW) and low-power vibrothermography technique for detecting debonding defects in CFRP-strengthened steel structures, optimizing excitation-frequency selection using the Sweep Frequency–Nonlinear Response (SFNR) method. Their approach enhances detection sensitivity and defect quantification while reducing complexity through a shared excitation source. A key innovation is the use of an air-coupled ultrasonic transducer, enabling non-contact detection and minimizing direct coupling effects. The study employs fast Fourier transform filtering and Otsu’s segmentation for thermal image processing, improving defect contrast and enabling automatic defect region extraction and size quantification. While their methodology was applied to debonding defects in CFRP-steel interfaces, it could also be extended to BVID detection in composite materials or artificial delamination studies. Other advanced feature-extraction techniques and other image-segmentation techniques could also be investigated that may improve the accuracy of the defect quantification.

Low-power vibrothermography has shown great potential for defect detection and quantification, particularly in composite materials. While advancements in analytical modeling and image-processing techniques have improved defect characterization, challenges remain in accurately quantifying defect depth, especially under low-power excitation. The integration of advanced signal processing, adaptive frequency selection, and hybrid NDT techniques offers promising directions for improving detection sensitivity and accuracy. Future research should focus on refining defect-depth quantification and exploring more robust feature-extraction and image-segmentation techniques, such as deep learning-based image processing, to enhance defect assessment in complex structures.

### 3.2. Defect Quantification Using Optical Thermography

This section will examine the application of optical thermography techniques for defect quantification. The effectiveness of this technique in quantifying defect size and depth will be analyzed, particularly in composite structures and aerospace applications. The discussion will highlight recent advancements, key challenges, and potential improvements in optical thermography for defect characterization. Special attention is given to heat conduction models, signal-processing techniques, and limitations in current methodologies.

#### 3.2.1. Heat Conduction Models for Defect Quantification

To better understand heat diffusion and defect quantification, several analytical models have been developed. One of the foundational models, proposed by Lau et al. [75], describes the surface-temperature evolution of a homogeneous specimen while considering subsurface defects as a semi-infinite air gap at depth *d*:

(18) $$T\left(0,t\right)=\frac{Q}{\sqrt{\pi \rho ckt}}\left[1+2\sum_{n=1}^{\infty }R^{n}e^{-\frac{{\left(nd\right)}^{2}}{4\alpha t}}\right]$$

Extending from Equations (7) and (8), this equation models surface-temperature variations after a pulsed thermal excitation. The first term represents the temperature decay in a homogeneous solid following a Dirac delta pulse of thermal energy *Q*. The second term accounts for thermal reflections at the defect interface, where *R* is the reflection coefficient, and multiple reflections are considered through the summation term.

To improve upon this model, Almond et al. [76] introduced a finite defect model that incorporates lateral heat-diffusion effects at the defect’s boundary:

(19) $$T\left(0,t\right)=\frac{Q}{\sqrt{\pi \rho ckt}}\left[1+2\sum_{n=1}^{\infty }R^{n}e^{-\frac{{\left(nd\right)}^{2}}{4\alpha t}}\right]\cdot \left(1-e^{-\frac{{\left(D/2\right)}^{2}}{4\alpha t}}\right).$$

This modification considers the defect as a finite-diameter flaw with diameter *D* and depth *d*, accounting for lateral heat diffusion, which influences surface-temperature distribution.

Building on this concept, Manohar et al. [99] developed a three-dimensional heat-conduction model to analyze heat flow around finite-sized defects in quasi-isotropic composite materials. The model calculates the excess surface temperature over a defective area compared to a defect-free region:

(20) $$\begin{array}{c} \begin{array}{cc} T_{e} & =\frac{ab\delta }{h_{1}h_{2}d}T_{0}\left(1+\sum_{n=1}^{\infty }\frac{2h_{1}}{n\pi a}\sin\left(\frac{n\pi a}{h_{1}}\right)e^{-t\alpha {\left(\frac{n\pi }{h_{1}\beta }\right)}^{2}}\right)\\ \; & \;\times \left(1+\sum_{m=1}^{\infty }\frac{2h_{2}}{m\pi b}\sin\left(\frac{m\pi b}{h_{2}}\right)e^{-t\alpha {\left(\frac{m\pi }{h_{2}\beta }\right)}^{2}}\right)\\ \; & \;\times \left(1+\sum_{k=1}^{\infty }2{\left(-1\right)}^{k}e^{-t\alpha {\left(\frac{k\pi \beta^{2}}{d}\right)}^{2}}\right). \end{array} \end{array}$$

Here, *ɑ* = ($K_{p}^{2}$ *K_z_*)^1/3^ represents the effective thermal diffusivity, and *β* =($\frac{K_{z}}{K_{p}}$)^1/6^ is the thermal conductivity ratio. *d* denotes the defect depth from the surface, and *t* is the time after the heat pulse, where $K_{z}$ and $K_{p}$ represent through-thickness and in-plane thermal conductivities, respectively. *a* and *b* define half the length and width of the defect, while $h_{1}$ and $h_{2}$ define half the length and width of the heated region.δ represents constant absorption thickness, and $T_{0}$ is the initial heat-pulse magnitude. This 3D heat-conduction model improves defect quantification by incorporating material anisotropy, heat-diffusion directionality, and thermal conductivity variations.

#### 3.2.2. Lateral Defect Quantification on Composite Material Using Optical Thermography

Lateral defect quantification is a crucial aspect of defect characterization, as it provides information on the size and spatial extent of a defect within a composite material. Optical thermography techniques, particularly pulsed thermography, have been widely used to extract lateral defect dimensions by analyzing thermal contrast, phase analysis, and image-processing techniques. This section will review various methodologies proposed in the literature for lateral defect quantification. In quantifying the defect size, Almond et al. [100,101] proposed the estimation of the size of the defect by using the Gaussian full-width half-maximum (FWHM) method. The estimation of the defect size is determined by drawing a line profile across the defective region, where the temperature distribution follows a Gaussian pattern, and measuring the full width at half maximum (FWHM). However, since FWHM decrease over time, obtaining measurements at an early stage yields more accurate results [102,103]. Zhu et al. [104] introduces the Temperature Integral Method (TIM), which determines defect size by integrating temperature values of each pixel across an area of defect to estimate the defect sizes. The defect size is given by:

(21) $$D\left(i\right)=\frac{T_{\mathrm{sum}}-LB}{\Delta T}$$

where $T_{\mathrm{sum}}$ = total summed temperature values along the profile line; LB is the background temperature contribution from the non-defective area; $\Delta T=T_{d}-T_{s}$ stand for the temperature difference between defective ($T_{d}$) and non-defective ($T_{s}$) regions. To ensure accurate measurement, TIM finds the characteristic time ($t_{i}$) at which the defect size should be measured:

(22) $$t_{i}=\frac{0.0031}{\alpha }{\left(\frac{T_{\mathrm{sum}}-LB}{\Delta T}\right)}^{2}$$

where α is the thermal diffusivity of the material. Unlike FWHM, which estimates defect size at an early stage of heating to minimize errors, TIM focuses on finding the precise moment (characteristic time) when defect size can be calculated most accurately. The paper also suggests combining the TIM method with the thermal signal reconstruction (TSR) method. However, TIM requires knowledge of the material’s thermal diffusivity (α), which may limit its practical application.

Several researchers use the image-processing technique combined with the image-segmentation technique to estimate the defect size of the specimens. Wang et al. [105] conducted a comparative study between long-pulse thermography, pulsed thermography, and step-heating thermography for the detection of flat-bottomed hole defects in GFRP and CFRP. Their study involved Absolute Thermal Contrast (ATC), thermographic signal reconstruction, Phase Fourier Analysis, and PCA’s image-processing technique. The study reveals that the combination of LPT and PCA demonstrated the highest defect-detection capability, particularly for deep-seated defects. The study relies on artificial defects (flat-bottomed holes), which may not fully represent real-world damage, such as delaminations and impact damage, limiting its applicability. Rajic et al. [106] applied principal component thermography to inspect the artificial delamination on the AS4/3501 composite laminate specimen. Based on their experiment, the second PC provides a useful basis for the estimation of flaw depth. Ardebili et al. [107] employed optical thermography to detect controlled defects in sandwich carbon-fiber composites, where defects were intentionally introduced into the carbon sheets, resin layers, and core materials. To enhance defect detection, PCA image processing was applied, improving contrast and noise suppression. However, the findings indicate that the technique struggled to detect defects located within the core and inner resin layers, particularly those embedded in deeper regions. The study suggests that future research should explore alternative image-processing techniques. The variant of PCA, such as robust PCA or sparse PCA, could be combined with optical thermography to investigate the effectiveness of this technique in detecting the defect located in the core of the sandwich panel.

Despite the widespread use of PCA in data-processing and dimensional reduction techniques, PCA has several limitations, such as being challenging to interpret because a principal component is a linear combination of all the original variables [108]. Thus, improvements are required for better results in detecting the defect through image sequence data. Several researchers proposed the improvement of the PCA methods. Tang et al. [109] introduced the Markov-PCA algorithm to improve the image’s SNR. In their study, it was found that window position affects Markov-PCA processing. Further improvement has been achieved by combining Markov-PCA with neural network theory to analyse the thermography thermal images [110]. The combined algorithm can quantify the depth and diameter of debonding defects on the thermal barrier coatings (TBCs). The study conducted by Tang et al. [110] focused solely on artificial circular defects and found that a decrease in the diameter–depth ratio will decrease the prediction error of the defect depth and defect diameter. When dealing with large, high-dimensional data sets and real-time video streams, Yousefi et al. [111] proposed candid covariance-free incremental principal component analysis (CCIPCT), a technique that does not require a covariance matrix and singular value decomposition (SVD) calculations. CCIPCT enables the rapid processing of input data within an online framework by dynamically updating eigenvalues and eigenvectors with each new data point [112]. A study conducted by Yousefi et al. [111] confirms that principal component thermography provides better contrast response compared to CCIPCT. However, CCIPCT significantly reduces computational expenses, making it advantageous for handling large, high-dimensional real-time data and enabling online processing capabilities. Therefore, CCIPCT may not be the best choice for applications requiring precise defect quantification and high contrast reliability. Thus, using CCIPT to quantify the defect size is not recommended.

Sparse principal component analysis (sparse PCA) or sparse principal component thermography (sparse PCT) is another extension of traditional PCA that addresses the interpretability challenges of PCA by generating principal components with sparse loadings [108]. Several studies attempt to apply sparse PCA to the thermography data [113,114,115]. Yousefi et al. [113] evaluate the sparse PCT for subsurface defect-detection accuracy in composite material by comparing it against other decomposition methods such as principal component thermography (PCT), CCIPCT, and non-negative matrix factorisation (NMF). Their findings revealed that sparse PCT demonstrated superior defect-detection capabilities for composite material defect images taken by the square-pulse thermography method. This finding is consistent with the study conducted by Wu et al. [114], which also concluded that sparse PCT is more interpretable than the PCT method for detecting defects in CFRP using pulsed thermography. Several advancements in sparse-PCA techniques have been proposed to enhance defect detection in thermographic data. An improvement involves applying data reconstruction methods before implementing SPCA. Jie et al. [116] introduced the sparse moving-window principal component thermography (SMWPCT) approach, where a preprocessing technique called the moving-window method is applied to reorganise thermal data. This method segments the data into smaller, overlapping subsets, capturing both temporal and spatial relationships before applying SPCA for defect detection. SMWPCT is well suited for dynamic thermal processes, enabling the detection of defects that evolve over time. Liu et al. [117] proposed sparse structural principal component thermography (S2PCT), which involved a shift-sampling augmentation technique to reconstruct thermal image data with specific sliding windows. This method reorganises the spatial structure of the thermal images by capturing local pixel relationships within each image. Subsequently, the reconstructed data are processed using sparse PCT to highlight defect signals while reducing noise and non-uniform background effects. Based on the study [117], it was found that S2PCT got a higher signal-to-noise ratio compared to SMWPCT when detecting defects on CFRP specimens containing the nine subsurface defects. Lou et al. [118] proposed spatial information-based PCA (SIPCA) to improve on traditional PCA by incorporating spatial relationships between pixels, which are often neglected during image-data vectorisation, enabling more accurate fault detection and localisation.

To address the limitation of the small data set, Liu et al. [119] proposed generative principal component thermography (GAN-PCT), which integrates data augmentation using generative adversarial networks (GANs) that generate synthetic thermal images with traditional principal component thermography. Liu et al. [120] further advanced this approach by introducing the generative-kernel principal component thermography (GKPCT) method. This method integrates the spectral normalised generative adversarial network (SNGAN), an enhanced GAN method, with kernel PCT to develop a GKPCT. As GAN and SNGAN are deep learning-based technique, its implementation requires significant computational resources and extended training time compared to traditional PCA.

Schölkopf et al. [121] introduced kernel principal component analysis (KPCA) as a groundbreaking extension of PCA to capture nonlinear relationships by mapping data into a high-dimensional feature space using kernel functions. KPCA has been applied in thermographic defect detection [115] and face recognition [122]. Based on this method, Jiang et al. [123] proposed parallel principal component analysis and kernel principal component analysis (KPCA) to solve the issues of mixed data relationships, where some variables exhibit linear relationships and others exhibit nonlinear relationships. Miao et al. [124] proposed adaptive KPCA (AKPCA) for fault detection in nonlinear chemical processes, addressing the limitations of traditional KPCA in handling mutation features. Although the implementation of the traditional KPCA on thermography image data has been studied, its variant’s application in identifying the defect in the composite material has not been studied yet. Future studies will adopt this technique in detecting the various defects of composite such as delamination and BVID.

To handle situations involving outlier or corrupted observations, Candés et al. [125] introduced robust principal component analysis (RPCA), which separates a data matrix into a low-rank component and a sparse component. Several variants of RPCA have been developed. Ma et al. [126] offer the reviewed variants of this RPCA. So far, this RPCA has been applied to detect the defect in the aluminium sample and stainless-steel sample using the eddy-current pulsed thermography technique [127] and to detect the defect in CFRP using the pulsed thermography technique [24]. The study of [24] revealed that the time required to process the data is higher than PCT, as the trade-off in improving defect detection. Another area for future investigation could involve the application of robust PCA in detecting the BVID on the composite using low-power vibrothermography.

#### 3.2.3. Defect-Depth Quantification on Composite Material Using Optical Thermography

While lateral defect estimation has been widely studied, accurately determining the depth of a defect presents additional challenges due to anisotropic heat diffusion and non-uniform defect geometries. Wang et al. [128] propose gaussianization transformation to improved the traditional defect-depth estimation method for pulsed thermography, specifically for CFRP materials. Traditional models assume that defect depth (*d*) follows a quadratic relationship with the peak contrast time ($\tau_{m}$). The peak contrast time is defined as the moment when the temperature contrast (ΔT) reaches its maximum [75,128,129]:

(23) $$\tau_{m}=\frac{2d^{2}}{\alpha }$$

where *d* is the defect depth and α is the thermal diffusivity. The study includes experimental testing on CFRP specimens with flat-bottom hole defects and Teflon-layered debonding defects, showing that their method improves the accuracy in determining the defect depth. Future studies may explore the effectiveness of this approach in estimating the depth of BVID, which presents additional challenges due to its irregular and complex thermal response.

Research conducted by Castanedo [80] presents a method for defect-depth retrieval across multiple materials using Fast Fourier Transform (FFT)-based phase analysis, known as pulsed-phase thermography (PPT). The pulsed-phase thermography was introduced by Maldague et al. [130,131]. Their approach relies on identifying the blind frequency $f_{b}$, which correlates with defect depth through thermal diffusivity and diffusion length calculations. The defect depth *z* is estimated using the following equation [80]:

(24) $$z=C_{1}\cdot {\left(\frac{\alpha }{\pi f_{b}}\right)}^{0.5}$$

where *z* is the defect depth, $C_{1}$ is an experimentally determined calibration constant within the range $1.5<C_{1}<2.0$, α is the thermal diffusivity of the material, and $f_{b}$ is the blind frequency. To improve depth-retrieval accuracy, the study emphasizes optimizing sampling intervals Δt and truncation windows w(t) to reduce noise and maximize phase contrast. The paper provides a systematic strategy for selecting these parameters. Unlike studies that focus exclusively on flat-bottom hole defects, this research also examines artificial delaminations and defects on the curved samples, demonstrating its adaptability to more complex structures. While Castanedo [80] tested artificial delaminations, D’Accardi et al. [132] investigated the stringer assy made of CFRP that has the real defect with non-uniform geometry using pulsed-phase thermography. The results were validated using the phased array method, and the study found that when the calibration constant $C_{1}$ was set to 2, it estimated a defect depth of approximately 1.2 mm. An alternative method for defect-depth estimation was proposed by Xu and Hu [133], where a Gated Recurrent Unit (GRU)-based deep learning model was implemented in active Infrared Thermography to recognize defect depth. The study conducted experiments on polymethyl methacrylate (PMMA) specimens with flat-bottom hole defects, employing PCA to reduce noise and redundant information before feeding it into the GRU network. The results showed that the PCA-GRU model outperformed a standard Backpropagation (BP) neural network. In the future, studies should carry out this approach for composite material with variation in the defect type and shape.

#### 3.2.4. Simultaneous Quantification of Lateral and Depth Defects Using Optical Thermography

Instead of only dealing with lateral defect quantification or defect-depth quantification, several researchers are working on both lateral defect quantification and defect depth quantification using optical thermography. Wei et al. [134] enhanced defect-depth estimation in pulsed thermography by introducing a normalization factor *N* that accounts for defect size (p=D/d) and material thickness (v=l/d), addressing the limitations of the classical peak slope time (PST) method [135]. The classical PST approach assumes one-dimensional heat conduction, leading to depth-estimation errors, particularly for large defects or thick materials. It estimates defect depth as:

(25) $$d_{\mathrm{uncorrected}}=\sqrt{2.71\alpha t_{\mathrm{PST}}}$$

where $t_{\mathrm{PST}}$ is the peak slope time, and α is the thermal diffusivity. The proposed method modifies this equation by incorporating *N*, determined experimentally from reference samples, to correct for lateral heat-diffusion effects:

(26) $$d_{\mathrm{corrected}}=\sqrt{\frac{2.71\alpha t_{\mathrm{PST}}}{N}}$$

A 5th-order polynomial model, developed from flat-bottomed hole (FBH) defects, enables the prediction of *N* for new defects by first determining the defect size. To determine the defect size, they use the image-processing technique proposed by Wei et al. [136]. This approach significantly improves accuracy, reducing depth-estimation errors from 24 percent (PST) to 5–7 percent, as validated through finite-element simulations and thermographic experiments. However, its reliance on FBH defects may limit its applicability to real-world defects like delaminations and BVID. D’Accardi et al. [137] present a structured methodology for estimating defect size and depth using PCA and thermal contrast analysis. Generally, the process begins with thermal data acquisition from a flat-bottom hole GFRP reference sample using pulsed thermography inspection, followed by pre-processing and temperature difference evaluation. The thermal sequence is then analyzed using principal component thermography to extract the most relevant eigenvalue orthogonal function. The optimal truncation window is determined by re-processing the thermal sequence frame by frame (changing the size of the truncation window). The best EOF for defect identification is selected, and Signal Background Contrast (SBC) is computed. A linear correlation between defect aspect ratio (D/t) and contrast is established using the reference sample, forming a calibration curve. This equation is then applied to real GFRP defects to estimate defect depth based on known diameters. The diameter of the defect could be determined by conducting binerization. Finally, ultrasound testing (UT) is used to validate the estimated depths, confirming the method’s accuracy. The detailed procedure can be found in [137]. This methodology offers a repeatable and quantitative approach for defect characterization, with potential applications for CFRP and other composite materials. However, a new set of reference samples would need to be manufactured, incorporating variations in fiber orientation and defect types.

## 4. Discussion

### 4.1. Challenges and Limitations in Thermographic Defect Quantification

Thermographic techniques have demonstrated significant potential for detecting and quantifying defects in composite materials, particularly CFRP. However, current methodologies often operate under idealized conditions that do not always translate effectively to real-world applications. This section discusses key challenges in thermographic defect quantification, highlighting gaps in the research and areas requiring further development.

This review paper compares the development of methods between vibrothermography and optical thermography. It can be observed that optical thermography has progressed further than vibrothermography in quantifying lateral defects, particularly in composite materials like CFRP. Evidence from this review indicates that optical thermography integrates experimental data with computer-vision techniques more extensively than vibrothermography. In vibrothermography, conventional image-processing methods are predominantly used, whereas optical thermography integrates more advanced techniques such as the variants of PCA. Moreover, optical thermography has incorporated deep learning-enhanced image processing, a step not yet widely evident in vibrothermography. The combination of the low-power vibrothermography with the deep learning technique is quite rare. To the best of the author’s knowledge, only one journal discusses the combination of the vibrothermography and the deep learning technique in detecting the defect on the BVID on the CFRP material [18]. The implementation of statistical image-processing techniques and a deep learning approach in analysing the data from low-power vibrothermography should be further investigated. It should be noted as well that another challenge of low-power vibrothermography is quantifying the defect depth, particularly in composite material.

Most studies evaluating thermographic techniques rely on standardized specimens with artificially induced defects, where the size and depth of defects are precisely known beforehand. While these setups provide valuable validation for new methodologies, they fail to reflect the complexities of real-world damage, such as BVID. Santo et al. [87] explored the detection of real-life defects using long-pulse thermography, providing insight into the challenges of assessing naturally occurring defects. However, the capability of low-power vibrothermography in detecting real-life defects remains largely unexplored and requires further investigation.

Another major limitation of existing research is the dominance of flat, square-shaped specimens in laboratory testing. These specimens provide an even surface for transducer placement, allowing for optimal ultrasonic excitation and heat diffusion. However, in real-world applications, composite structures such as aircraft fuselages, automotive panels, and wind-turbine blades are highly curved and three-dimensional. This geometric complexity presents significant challenges for vibrothermography, particularly in ensuring consistent ultrasonic coupling and uniform excitation. Several studies have attempted to detect defects on a non-flat specimen, like Liu et al. [138], who developed modified transducer designs specifically for sucker rod-shaped specimens, demonstrating the feasibility of customizing transducers to match complex geometry-adaptive transducers that can conform to non-flat surfaces. Solodov et al. [53] introduced air-coupled ultrasonic excitation techniques that eliminate the need for direct contact between the transducer and the specimen. The effectiveness of the low-power vibrothermography approach to different geometries of the sample needs to be studied.

In aerospace applications, composite structures are commonly coated with paint for both aesthetic and protective purposes. However, this presents a significant challenge for thermographic inspection, as removing the paint layer is neither practical nor efficient. Paint alters the thermal diffusion properties of the material, which can reduce the sensitivity of infrared cameras in detecting subsurface defects. Although a study by Grosso et al. [139] demonstrated that pulsed thermography is effective in detecting corrosion beneath coatings and evaluating adhesion degradation in anticorrosive composite materials, the effectiveness of low-power vibrothermography in detecting and quantifying defects concealed beneath coatings or paint remains to be investigated. Future studies should also investigate whether data-processing techniques such as thermal signal reconstruction or machine learning-based contrast enhancement can compensate for the interference caused by paint layers.

High-resolution infrared cameras are essential for precise defect quantification, but they are expensive and not widely accessible. To make thermographic inspection more practical for industry applications, low-cost alternatives must be explored. Anwar et al. [19] demonstrated that low-cost, uncooled microbolometer infrared cameras combined with image-segmentation techniques could detect defects in glass-fiber-reinforced polymer materials using long-pulse thermography. The effective application of the low-cost thermal camera in low-power vibrothermography also needs to be studied.

Studies have shown that detecting defects at depths greater than ten millimeters is difficult, and for materials with a thickness of twenty millimeters or more, the effectiveness of low-power vibrothermography must be systematically evaluated. Tromaras et al. [88] investigated thick monolithic CFRP structures using step-heating thermography, revealing significant limitations in heat penetration depth. Addressing this issue may require optimized excitation methods or a combination of the vibrothermography with an advanced image-processing or deep learning approach, or the other NDT technique [98].

### 4.2. Future Directions

Future research should investigate the capability of vibrothermography for detecting real-life defects in composite materials, moving beyond standardized specimens containing artificial defects. Developing adaptive transducer designs, such as those proposed for sucker rod-shaped specimens by Liu et al. [138], could improve defect detection in complex geometries. Air-coupled ultrasonic excitation should be evaluated to eliminate direct transducer contact, which may be applied for curved composite structures.

The influence of paint layers on defect detection should be studied further, and researchers should explore image-enhancement techniques to compensate for signal loss. Investigating the feasibility of using low-cost microbolometer infrared cameras in aerospace-grade CFRP inspections would also provide valuable insights. Future studies should evaluate the effectiveness of low-power vibrothermography for defect detection in thick composite structures which have a thickness of more than 10 mm.

To advance thermographic techniques for defect quantification, research must shift towards real-world applications by addressing non-flat geometries, painted surfaces, cost-effective solutions, and deeper defect detection. By integrating adaptive transducer technology, non-contact ultrasonic excitation, machine learning for data enhancement, and hybrid nondestructive testing approaches, vibrothermography may become a more robust and industry-relevant inspection method.

## 5. Conclusions

Thermographic techniques, particularly vibrothermography and optical thermography, have demonstrated significant potential for detecting and quantifying defects in CFRP composites. This review highlights advancements in these methods, emphasizing the role of image processing in improving defect characterization. Optical thermography, particularly pulsed thermography, has seen extensive development in integrating machine learning and deep learning techniques for enhanced defect detection. In contrast, vibrothermography, despite its promising capabilities, has yet to benefit from similar advancements in image processing.

A key research gap identified in this review is the limited integration of advanced computational methods, such as PCA variants and deep learning, in vibrothermography for defect quantification. Addressing this gap presents an opportunity to improve the accuracy and reliability of defect assessments, particularly for lateral defects such as delaminations and BVID.

Future research should focus on bridging the methodological differences between optical and vibrothermographic techniques by incorporating modern image-processing algorithms in vibrothermography. Additionally, further exploration of low-power vibrothermography and its integration with advanced computer-vision techniques could enhance defect-detection capabilities while minimizing energy consumption. Advancements in this field will contribute to safer and more reliable composite structures across industries, reinforcing the role of thermographic methods in non-destructive testing.
